# Voluntary Hospitals in Relation to Social Service Work

**Published:** 1914-07-04

**Authors:** Herbert Shaw

**Affiliations:** Sheriff of Newcastle-upon-Tyne


### THE BRITISH HOSPITALS ASSOCIATION.

### FIFTH ANNUAL CONFERENCE AT NEWCASTLE-ON-TYNE, JUNE 18 to 20, 1914.

SECOND DAY'S PROCEEDINGS.

The proceedings were resumed on Friday morning, June 19, and the chair was again occupied by Councillor W. J. Sanderson, the Vice-Chairman of Newcastle Infirmary House Committee. There was again a good attendance.

### VOLUNTARY HOSPITALS IN RELATION TO SOCIAL SERVICE WORK.

**By COUNCILLOR HERBERT SHAW, B.A., Sheriff of Newcastle-upon-Tyne.**

The following is a condensed report of Councillor Shaw's paper, the reading of which was the first item of the proceedings on Friday morning:—

Students of social service during the last few years cannot fail to have been impressed by its growing importance. A somewhat idealistic attempt made a few years ago to supplement the medical work of the hospitals in a limited way has to-day become an integral part of the work of healing. It is now not only recognised as a valuable supplement, but bids fair ere long to become as important as hospital work itself.

The reason is obvious. The present generation has discovered this, that if the work of healing is to be of any avail the whole man must be considered, in his moral and social as well as his physical aspect. If the principles of modern scientific medicine are to be applied wisely and effectively, the problems of bad housing conditions and poverty must be dealt with and ignorance combated. Hence the idea has grown upon us that the physician and social worker must of necessity join hands if their work is to be worth the doing.

There is undoubtedly a great deal of work in the hospital that the doctor cannot do himself. For example, he has not the time to instruct his patients in hygiene, to teach men new sets of habits. Yet without hygiene much of his skill is useless, and without skilful fitting to the individual's needs hygienic rules are simply worthless; and therefore, if his work is to be really effective, "someone" must step in and do what the doctor himself cannot do.

### The Social Service Movement.

Then, too, as a rule, the search for better health involves expenditure of money. In consumption, heart disease, and other serious cases someone has to find the money if the doctor's orders are to be carried out. He himself certainly cannot be expected to find the money. At the same time, he feels the mockery of ordering, say, for one patient a diet which he cannot buy, for another a holiday which he cannot possibly afford, for another an outdoor life which he knows he cannot possibly enjoy, and so on almost indefinitely.

To make the doctor's work worth while to himself and to the patient, it must therefore be done in conjunction with someone who has time and ability to teach hygiene and see that it is carried out, to study a patient's home conditions and report upon them, and to help to modify retarding conditions, whether they be financial, mental, or moral, which stand between the patient and recovery.

This "someone" is undoubtedly the social worker—the man or woman trained to think of the human being as a whole just as naturally as the physician concentrates attention upon a part. He it is who finds out what is missing of the essentials of human life in the patients dealt with, and who supplements the efforts of the specialist in the interests of healthy, living character.

In America social service of the nature which I have indicated has been a real thing for a considerable time past, and benefits from the customary excellent power of organisation for which our transatlantic cousins are not a little famous. For instance, the social workers at the General Hospital of Massachusetts are a highly trained and organised body, but at the same time only one of many similar groups of hospital social workers in America who are striving to make their contribution to the business of promoting the economy, efficiency, and humanity of the hospital a real thing. In the Pennsylvania University Hospital social workers, I may add, are even assigned to special groups of cases, whether in the ward or the dispensary.

### The Work of the Hospital Almoner.

The most important connecting-link in this chain of charity between the hospitals and the institutions is that of the hospital almoner. He or she is the one who draws together the doctors, the social workers, the charitable societies, and hospitals. He or she brings expert knowledge to bear on patients, and puts them into touch with that "someone" who does so much for the afflicted. Besides acting as a check on abuse, the almoner sees that the doctor's orders are carried out, and that they are made effective, by introducing the patient to some charitable society which is eager to do the work indicated—the work which must supplement the physician's labours if they are to be of any avail.

The Royal Victoria Infirmary in Newcastle has now, happily, got this connecting-link between hospitals and social service. The Lady Almoner, whose work commenced in January 1913, tells us in her report all about the co-operation between our greatest voluntary hospital in Newcastle-upon-Tyne and outside charitable agencies, so that it is unnecessary for me to do more than indicate it. Suffice it to say that she seems to have brought hospital patients most effectively into touch with almost every charitable agency of the district—such as the Charity Organisation Society, the Guild of Help, the Surgical Aid Society, etc.—that they may benefit to the full by the treatment given to them. Good work has also been done for the Maternity Hospital by social workers.

It would be impossible to mention all the numerous societies which are working in conjunction with our voluntary hospitals. Yet there is room for closer co-operation than at the present time exists. In this respect the almoner system is to be welcomed by local charities which are in combination with the Social Service of the Charity Organisation Society, the Guild of Help, Surgical

Aid Society, the Mothers' and Babies' Welcome Society, the Chest Hospital, Invalid Loan Society, the Convalescent Homes, the Cripples' Home, and the Blind School.

What the Social Worker Should Aim at.

The world is still faced with the ogre of "preventable disease"; for disease is not often nowadays the result of accident or of "hard luck." It is much more frequently the fruit of undesirable social conditions and bad habits. So that the work of our voluntary charities—whether they be social workers or hospitals—is the prevention of the evils against which they are fighting. And experience shows that co-operation in this, as in most other great undertakings, is the force which will bring about the best results. The closer the bond between the voluntary hospitals and the social worker the more effective will their work be. Without exaggeration we may say that such a linkage and brotherhood of helpfulness has no end, and that as far and as fast as we can readily achieve it we bring about the fulfilment of our ideals. Can anything be more inspiring than the thought of the endless chain of helpfulness I have tried to indicate?

The Social Service Organisation must be a teacher to the patient—and to all that portion of society of which he happens to be representative—and upon him it must impress the basic fact that to no small extent the betterment of his own condition is dependent upon himself, and upon his efforts so to change the character of his environment and the other social influences that play upon him as to allow him to actualise those best qualities of body, mind, and soul that are potential within him. It is in the hands of the thousands of voluntary workers all over the country to create that good understanding, and to give to the work their time, brains, and energies.

"Not as a ladder 'twixt earth and heaven, not as a witness to any creed,
But by simple service, simply given, to our own kind in their common need."

### THE DISCUSSION ON COUNCILLOR SHAW'S PAPER.

Mr. Kemp, of the Charity Organisation Society, said he was very glad indeed to have the opportunity of saying a few words on the very important subject which Mr. Shaw had addressed them on that morning. Mr. Thies had kindly invited him to do so because of his work in London and on the medical committee of the Charity Organisation Society. Mr. Shaw's contentions might be illustrated by a number of actual examples. He used to be secretary of a district of the Charity Organisation Society in London where they had very often to deal with three or four letters from almoners at hospitals every day, and had all sorts of difficulties to contend with. Examples taken from an almoner's report might illustrate very well some of the suggestions, particularly as to the importance of getting to what he very well called the background of a patient's life. If it was admitted that work of that kind should be done, what were the essentials? He suggested there were two, and the first was obviously goodwill. The work also of the various staffs, including those who were paid, was no less an instance of goodwill. Goodwill would not take them the whole way. They wanted besides goodwill a good deal of knowledge, experience, and training, if they were to do their work properly. In these days it took them all their time, only to refer to one thing, to keep up with Acts of Parliament that were continually coming along. They must know about these things, or they could not do their work properly. He believed that the time would come when any hospital which did not possess a social service department would be reckoned to be incomplete.

The Origin of the Inquiry System.

Mr. Conrad W. Thies said that he was very much interested in this phase of hospital work, and that when he first became secretary of the Royal Free Hospital he found that there was no regular system of inquiry among patients. About twenty years previously to that an inquiry had been made under the auspices of a special committee of which Sir William Ferguson had been the chairman, and which obtained the permission of the hospital authorities to make inquiry at the Royal Free Hospital into the circumstances of the patients in the out-patient department. The results of the inquiry were put into a report, which was printed and published and became the text for speakers for many years afterwards. It was proved that there was a large amount of abuse in respect of patients in voluntary hospitals in the out-patient department, because there was no regular system of inquiry into the means of patients. The result was that a philanthropic gentleman in London took up the challenge, and agreed to pay the expenses for three years of an almoner if they would accept one of the Charity Organisation to make inquiries. His committee without any hesitation consented, and it was agreed that the hospital should allow the almoner arranged by the Charity Organisation Society to come into the hospital and have proper facilities given her in the way of inquiries being made into the circumstances of the patients. They only made a stipulation that nothing was to be published in connection with the inquiries made without the hospital committee's consent. An experienced lady was appointed, and they did all in their power to facilitate her work, and it was found that the abuse was comparatively small. It was found that there had been eliminated a large number of unsuitable cases. The result was proved to be so satisfactory that a three years' arrangement was made between the Charity Organisation Society and their own committee at the hospital, and the work taken up and the expense defrayed.

An Almoner's Department.

The Charity Organisation Society assisted them by contributing towards the cost of the inquiry. The hospital took up the whole responsibility, and at present they employed two fully paid almoners, who had two paid assistants, and in addition they had the assistance of a certain number of ladies who voluntarily devoted their services free to this particular work in connection with the hospital. The result was that the patients had benefited in many ways. On their leaving they were given good and careful advice as to their personal habits and how they should attend to their children. In addition there were some cases referred to some agency where the work of the physician or surgeon could be properly followed up. In addition they came in at the hospital for a large sum of money to be devoted to patients after leaving the hospital. Some years ago he was in America with the British Association in Winnipeg. He had been at the General Hospital of Massachusetts, in Boston, which had been referred to by Mr. Shaw, and he had spent a couple of days in watching the social service work in operation there, and he had found it a most perfect system. It gave him many ideas that he was able to make use of when he came back in his own hospital. It was a magnificent system of social service, and was organised outside quite independent of the hospital, which gave every facility by supplying accommodation; but the whole expense was carried on by a separate organisation, which had the full run of the hospital and managed the whole business. The result was not only beneficial to the hospital, but also to the whole charitable work of the city of Boston. There was a preventing of overlapping by the system, and in a general way there was an economising effect over all the institutions.

Mr. J. Courtney Buchanan, of the Metropolitan Hospital, London, said he should like to deal with the positive and constructive side of the almoner's work. That had a more practical bearing on the paper on social service. It was his firm belief that it would be through the almoner's department that the hospital would gradually become more associated with the general public and the whole community, because they would gradually see the educational value of hospitals quite apart from the training of the nurses and doctors, and the advance made in medical science. Quite apart from that, however, the social education of the poor people coming to the hospital was of very great value to the community. The Poor-Law Commission report drew attention to the importance of fighting disease in the patients' own homes. The National Insurance Act was practically the only outstanding measure that had taken into account that report. Any number of orders and administrative alterations had been made in consequence of the findings of the Commissioners, but the National Insurance Act was the one definite Parliamentary measure which had so far been passed to deal with this position. While the London County Council was dealing with the treatment of the disease, the borough councils were working for the out-patient treatment near patients' own homes. The borough councils had direct instructions from the Local Government Board to link up their organisation with the hospitals, and at the Metropolitan Hospital they had already arranged for the establishment of a tuberculosis dispensary. He believed that through this work there would be another wave of philanthropy, and that they would have the opportunity of getting over their difficulties in respect of hospitals generally about which they had heard so much at the Conference on the previous day.

Mr. Howard J. Collins referred to the portion of Mr. Shaw's paper stating that already it had been found that the best results came from the work of the organisation as accruing from the combination of movements. That should be by co-ordination rather than by the co-operation of the hospital with outside agencies. He thought that hospitals had already enough work to do, and many of them too much. What should be done—and many of them tried to do it—was to refer these cases to the proper channel, and not attempt to interfere in any way. When an infectious case broke out they notified the official of the sanitary authority, and that authority removed the case and dealt with it. They did not undertake to discuss with them how to treat the case. They did not attempt to say how many doses they should give the patient, but they said, "Here is a case which we will not undertake to deal with; please take it from us." It seemed to him that social service ought to be on that line. He thought that they had completed their work when they handed over the case to the proper organisation, and ensured that that body was informed of the circumstances, and that they should deal with the case in the way that they thought best.

Mr. E. Stewart Johnson, of the Hospital for Sick Children, Great Ormond Street, London, said he should like to point out some of the limitations to the work of the almoner, but it must not be understood that he was speaking against the almoner's system. It was the object of the almoner's system to supply what the medical side of the work was unable to do. It was to see that the medical treatment was not lost. In London there was a large proportion of the hospital patients for whom it was difficult to provide. In the report of the Poor-Law Commission by Miss Roberts it was shown that at least two-fifths of the patients were practically Poor-Law cases. At the hospital where he came from it was much the same thing. About 40 per cent. of the patients were too poor to provide for medical treatment, and it was almost impossible for the almoner to procure sufficient help to enable them to benefit by medical treatment. They could obtain grants for food from the Poor Law, yet at the same time there was no doubt many of them were underfed, improperly clothed, and improperly housed, and it was quite out of the power of the almoner to procure help for more than any temporary crisis. What the hospital had to do under the circumstances it was very difficult to say. One could not refuse to treat such cases. At the same time, it was very difficult for a body which depended for its support upon the public to point out what it was not able to do. They had to go on treating these people, but it was necessary that they should be helped in some other way, and he would leave to others to find out whether it should be by a minimum wage or by an endeavour to decasualise labour. Until that took place, a very great deal of the money spent by hospitals would be wasted.

